# Emerging Concepts in Vector Development for Glial Gene Therapy: Implications for Leukodystrophies

**DOI:** 10.3389/fncel.2021.661857

**Published:** 2021-06-22

**Authors:** Georg von Jonquieres, Caroline D. Rae, Gary D. Housley

**Affiliations:** ^1^Translational Neuroscience Facility, Department of Physiology, School of Medical Sciences, UNSW Sydney, Sydney, NSW, Australia; ^2^Neuroscience Research Australia, Randwick, NSW, Australia

**Keywords:** leukodystrophies, gene therapy, glia, adeno-associated virus, hematopoietic stem cells

## Abstract

Central Nervous System (CNS) homeostasis and function rely on intercellular synchronization of metabolic pathways. Developmental and neurochemical imbalances arising from mutations are frequently associated with devastating and often intractable neurological dysfunction. In the absence of pharmacological treatment options, but with knowledge of the genetic cause underlying the pathophysiology, gene therapy holds promise for disease control. Consideration of leukodystrophies provide a case in point; we review cell type – specific expression pattern of the disease – causing genes and reflect on genetic and cellular treatment approaches including *ex vivo* hematopoietic stem cell gene therapies and *in vivo* approaches using adeno-associated virus (AAV) vectors. We link recent advances in vectorology to glial targeting directed towards gene therapies for specific leukodystrophies and related developmental or neurometabolic disorders affecting the CNS white matter and frame strategies for therapy development in future.

## Leukodystrophies – A Complex Group of Rare Diseases

Leukodystrophies *(leuko*, white; *dystrophy*, wasting) are a heterogeneous group of genetically determined disorders primarily affecting white matter in the central nervous system (CNS). Although each individual leukodystrophy is a rare disease, the collective overall incidence has been reported to range between 1: 5000 and 1:100.000 live births, rivaling the incidence of acquired white matter diseases including multiple sclerosis (Heim et al., 1997; Bonkowsky et al., 2010; Vanderver et al., 2012).

The term leukodystrophy was first established almost a century ago and until the mid-1980’s considered to primarily affect myelin or oligodendrocytes directly (Bielschowsky and Henneberg, 1928; Morell, 1984). Advances in diagnosis through magnetic resonance imaging (MRI), whole exon and whole genome sequencing have resolved broader non-myelin components of this white matter pathophysiology. In recognition of this the term leukoencephalopathy has partially supplanted leukodystrophy, but traditionally includes acquired disorders that do not have a hereditary cause. Despite diagnostic advances, in ∼ 25 % of diagnosed leukodystrophies, the genetic cause remains enigmatic (Vanderver et al., 2016, 2020). Thus, an exhaustive definition and classification of leukodystrophies and leukoencephalopathies is ongoing. Van der Knaap et al. have recently postulated a new categorization of leukodystrophies in the clinical setting and proposed a novel, useful classification of leukodystrophies based on cellular pathology (Vanderver et al., 2015; Kevelam et al., 2016; van der Knaap and Bugiani, 2017; van der Knaap et al., 2019). Following this classification leukodystrophies are grouped into classical *myelin disorders* that include hypomyelinating, demyelinating and vacuolizing leukodystrophies as well as *astrocytopathies*, *microgliopathies, leuko-axonopathies* and *leuko-vasculopathies* (van der Knaap and Bugiani, 2017).

While most leukodystrophies can manifest at any stage of life, there is usually an inverse correlation between the age of onset/diagnosis and disease severity. Particularly early-infantile onset leukodystrophies are typically associated with rapid progression and relentless, frequently fatal patient decline (Waldman, 2018; van der Knaap et al., 2019). However, not all leukodystrophies have a clear predictable course. Prognosis may depend on the specific mutation, age and severity at presentation and other genetic and environmental factors. More benign progression characterized by episodes of stability and even permanent improvement followed by recovery are regularly reported in some leukodystrophies (Köhler et al., 2018; van der Knaap et al., 2019).

Indisputably, advances in diagnosis and understanding of pathomechanisms have tremendous impact on management and treatment of the specific disease. Substrate reduction and pharmacological treatments generally target symptoms and have been covered in excellent reviews, but drugs that effectively treat the underlying disease and improve progression remain elusive (Adang et al., 2017; van der Knaap et al., 2019). Direct targeting of the underlying genetic basis of these diseases is therefore seen as a principal therapeutic strategy. This review will focus on the developing framework around gene therapies of these rare and debilitating genetic diseases.

## Current Treatment Approaches

In-depth knowledge of gene variants, associated pathomechanisms and prognosis is indispensable to make treatment decisions. The pathology of leukodystrophies range from mild to severe and often have a clear genotype – phenotype correlation. In more severe cases, the absence of pharmaceutical options for targeted treatment of CNS disorders have moved focus towards putatively curative gene and cell therapies. Due to the complexity of individual genetic causes and the limitations of different vectors, development has been lagging. It is becoming increasingly clear that there will not be a simple standardized solution. Therapy will need to be tailored and personalized.

In certain leukodystrophies, characterized by a build-up of cytotoxic metabolites in the brain, bone marrow transplant or allogenic hematopoietic stem cell transplantation (HSCT) can be very effective in attenuating disease progression. This has been the preferred treatment in pre-symptomatic X-linked Adrenoleukodystrophy (ALD), metachromatic leukodystrophy (MLD) and Krabbe disease patients over the past 30 years (Escolar et al., 2005; Musolino et al., 2014; Boucher et al., 2015; Page et al., 2019). The rationale behind HSCT in slowing or arresting disease progression is that microglia, hematopoietic cells of the myelomonocytic lineage can infiltrate the CNS following myeloablation and express the missing gene necessary for degradation of the cytotoxic metabolite. In addition, intrathecal injection of cord blood-derived oligodendrocyte-like cells (DUOC-01) that have been shown to express lysosomal enzymes are currently assessed in clinical trial (NCT02254863) for their potential as an adjuvant bridging the temporal gap between allogenic HSCT transplant and engraftment of cells in the CNS thus more rapidly preventing disease progression (Kurtzberg et al., 2015; Saha, 2018). Success of HSCT largely depends on pre- or very early symptomatic treatment and the availability of a human leukocyte antigen (HLA) compatible donor, with cord blood of a non-carrier sibling being the preferred source. However even where HLA-matched donors are available, graft failure, graft versus host disease and risks of severe infections associated with prolonged immunodeficiency following myeloablation and engraftment are common complications (Boucher et al., 2015; Bonkowsky et al., 2018; Hierlmeier et al., 2018).

## Gene Therapy Approaches

In the following section we provide a perspective on recent progress in the field. Broadly, gene therapy approaches can be divided into *ex vivo* and *in vivo* approaches, each using either viral or non-viral vectors for delivery of coding or non-coding therapeutic nucleic acids. Despite substantive progress in the field, which have expanded gene therapy strategies personalized approaches dependent on the patient, the specific disease, and its progression at treatment are key precepts.

## *Ex vivo* Gene Therapy

*Ex vivo* gene therapy builds on HSCT and largely overlaps with cell therapy in that patients’ own stem cells are extracted genetically modified via chromosomal integration of a therapeutic DNA, expanded, analyzed and reintroduced into the same patient. The advantage of this approach is that graft versus host disease and graft failure as well as other immune complications are largely eliminated (Staal et al., 2019). Pioneering this approach in a leukodystrophy, Nathalie Cartier and Patrick Aubourg’s group used this autologous CD34^+^ HSCT to successfully treat cerebral ALD in patients where no appropriate HLA – match could be obtained (Cartier et al., 2009). Following myeloablation, using a Busulfan or similar regimen, intravenous (IV) administered HSC differentiated into microglia-like cells following engraftment in the human CNS. Initially using murine leukemia virus based gammaretrovirus (γRV) vectors, followed by human immune deficiency virus-based lentivirus (LV) vectors, development of protocols and screening procedures have significantly improved the safety profile of hematopoietic stem cell gene therapy (HSC-GT) (Schmidt et al., 2007; Biffi et al., 2011). Both γRV and LV – based vectors integrate semi – randomly into the host genome producing stable replication and passage of the inserted therapeutic gene upon cell division but the corollary is the inadvertent risk of rare insertional mutagenesis – induced malignancies. While LV preferably integrate into transcriptionally active chromatin, γRV predominantly integrate in the vicinity of gene regulatory regions (Vranckx et al., 2016). Particularly γRV show a long terminal repeat (LTR) – mediated propensity for recurrent insertion into ‘hot spots’ frequently near transcription start sites of proto-oncogenes explaining the heightened risk profile of early γRV vectors over LVs vectors for gene therapy applications (Cattoglio et al., 2007). Transcriptionally active LTRs are the principal determinants of this insertion mediated genotoxicity (Montini et al., 2006, 2009), and development of self – inactivating LV and γRV achieved by deletions in the 3’ – LTRs that abolish intrinsic LTR promoter activity significantly enhanced the safety profile of these vectors (Zufferey et al., 1998).

Encouraging preclinical results employing self-inactivating LV vectors led to an open-label, phase II/III clinical trial involving autologous CD34^+^ via self-inactivating lentivirus vector – mediated integration of ABC transporter D1 (*ABCD1*) cDNA in the STARBEAM study (ALD-102) in X-ALD patients (Eichler et al., 2017). Latest reports from this study suggest that disease progression stabilized in 31 out of 32 enrolled patients with all 15 patients that have completed a two year follow up remaining free of major functional disabilities (Kühl et al., 2020). Similarly, following promising clinical safety and efficacy results demonstrating improvement of Gross Motor Function Measure in 20 MLD patients for up to eight years, Libmeldy, an arylsulfatase A (*ARSA*) HSC-GT for early onset MLD has recently received a marketing authorization by the European Medicines Agency (EMA) on the basis that the benefits outweigh the risks, becoming the third approved HSC-GT after Zynteglo^TM^ and Strimvelis^TM^ (Biffi et al., 2013; Sessa et al., 2016; Tucci et al., 2020).

At present, *ex vivo* HSC or HSC-GT is predominantly used in leukodystrophies caused by accumulation of toxic metabolites that are degradable by a therapeutic gene product expressed in microglia-like monocyte derived macrophages after CNS infiltration, which may be supported by DUOCs in future. As for other therapeutic approaches, therapeutic benefit of HSC transplantation for leukodystrophies is inversely correlated with CNS disease progression, likely because late HSC administration alone cannot restore irreversible damage from neuron loss.

For other leukodystrophies, cell-based gene therapies may be extended to direct injection of genetically modified stem or progenitor cells in the future (Osorio and Goldman, 2016). The potential of genome editing using Zinc – finger nucleases, transcription activator like effector nucleases (TALEN) and clustered regulatory interspaced short palindromic repeats (CRISPR) – Cas9 systems offers immense opportunity for targeted correction of disease-causing mutations (Ferrari et al., 2020). Although pre-clinically gene-editing was successfully employed in HSC-GT, further safety and efficacy studies will be conducted (Schiroli et al., 2017; Pavel-Dinu et al., 2019). *Ex-vivo* gene addition and gene editing are readily achievable employing non – viral approaches including gene – electrotransfer. A notable advantage of gene editing is the preservation of normal copy numbers and gene regulation (Pinyon et al., 2019; Wagenblast et al., 2019). A recent study has modified induced neural progenitor cells (NPCs) and induced oligodendroglial progenitor cells (OPCs) from Canavan disease (CD) patients to express human aspartoacylase (*ASPA*) via lentivirus mediated gene addition and TALEN mediated gene editing. Direct injection of either of these genetically modified induced pluripotent cells (iPCs) into a CD mice resulted in pronounced histological, pathophysiology and motor behavior improvement (Feng et al., 2020). Observations from allogenic transplantation of human CNS stem cells into four Pelizaeus-Merzbacher disease patients showed engraftment and focal production of donor derived myelin in the transplanted hosts’ white matter but detected an immune response and donor-specific HLA alloantibodies in half the patients (Gupta et al., 2012, 2019). Albeit holding promise, cell-based CNS gene therapies face significant hurdles to clinical translation.

## *In vivo* Gene Therapy

*In vivo* gene therapy relies on direct injection of the ‘naked’ or encapsulated therapeutic nucleic acid into the patient. While many virus derived vectors including recombinant adenovirus, LV, γRV, sindbis virus, poliovirus and herpes simplex virus have been trialed for CNS gene delivery, based on their superior safety and transduction efficiency, versatility and easy production credentials, recombinant adeno-associated virus (rAAV) has taken the center stage in the development of gene therapies for neurological disorders (Lentz et al., 2012; Ojala et al., 2015). Of the three AAV based therapeutics that have obtained regulatory approval by the European Medicines Agency (EMA) or U.S. Food and Drug Administration (FDA) since 2012, two target sensori-motor neural diseases. Luxturna^®^ (Voretigene neparvovec) has been developed for Leber’s congenital amaurosis using subretinal injection and Zolgensma^®^ (Onasemnogene abeparvovec) is directed at spinal muscular atrophy in children under the age of two years, utilizing intravenous (IV) delivery.

*In vivo* gene therapy for leukodystrophies requires therapeutic gene transfer to the CNS which is compromised by the skull, the blood – brain barrier (BBB) and cerebrospinal fluid (CSF) – brain barrier (CBB). Delivery methods for rAAV to the brain include direct intraparenchymal (IP) injection, intracerebroventricular (ICV), lumbar intrathecal (IT) intra cisterna magna targeted intrathecal (ICM) and IV injections as well as nasal delivery which have been addressed in excellent recent reviews (Hocquemiller et al., 2016; Piguet et al., 2017, 2020; Hudry and Vandenberghe, 2019). In the following section, we review the advances in understanding of AAV vector targeting to provide context for the likely dominance of such gene therapy vector strategies for treating leukoencephalopathies in the future.

## The Evolving Adeno-Associated Viral Vector Toolkit

### Natural Discovery of Adeno-Associated Virus for Use as Gene Therapy Vectors

AAV is a non-enveloped, replication-deficient virus of approximately 25 nm in diameter whose icosahedral AAV capsid harbors the single stranded (ss) DNA genome. Cell surface receptor binding, internalization, endosome escape and trafficking as well as aspects relating to immune escape and AAV production including stability and assembly are mediated through the capsid. As such the AAV capsid is the principal determinant of biodistribution, cell and tissue tropism and the site of interference with circulating neutralizing antibodies. The AAV capsid is a 60mer composed of three viral proteins VP1, VP2 and VP3 encoded in a single open reading frame, that assemble in a 1:1:10 ratio. Based on sero – reactivity, at least 13 human and non-human primate (NHP) AAV serotypes have been isolated, but seminal work from Guangping Gao and James Wilson’s lab identified more than 100 variants in NHPs in the early 2000’s and divided these naturally occurring AAVs into phylogenetic clades on the basis of functional and serologic similarities (Gao et al., 2004, 2005). Since then, AAVs have been isolated in different vertebrates, with many capable of cross-species transmission (Allison et al., 2013; Zinn and Vandenberghe, 2014). AAV serotypes isolated from natural sources generally have a broad tropism but often have cell type or tissue specific bias which varies depending on delivery route, target species, age of infusion and purification method (Klein et al., 2008; Zhang et al., 2011; Aschauer et al., 2013; von Jonquieres et al., 2013; Watakabe et al., 2015).

Comprehensive investigations of AAV tropism have been performed for select AAV serotypes and variants. Intraparenchymal AAV injection into the adult rodent brain using constitutive promoters to drive transgene expression suggests that most AAVs preferentially transduce neurons. Amongst others these include AAV1, AAV2, AAV5, AAV7, AAV8, AAV9, AAV.rh8, AAV.rh10, AAV.rh20, AAV.rh39 and AAV.cy5 with superior transduction efficiencies achieved by AAV1, AAV9, AAV.rh10. (Bartlett et al., 1998; Passini et al., 2003; Burger et al., 2004; Cearley and Wolfe, 2006; Lawlor et al., 2009; Castle et al., 2016). While transduction of astrocytes was observed for most serotypes, AAV8 and AAV.rh43 showed strongest astroglial tropism, with AAV8 also revealing some oligodendrocyte transduction (Davidson et al., 2000; Klein et al., 2008; Hutson et al., 2012). In the spinal cord AAV1, AAV5, AAV9 have shown strong neuronal tropism, but AAV8 was superior at targeting large diameter neurons (Jacques et al., 2012). It must be clarified, that exchanging the promoter driving transgene expression can completely change the expression profile of AAV vectors including towards oligodendrocytes, underscoring the broad tropism of AAVs in the CNS (Lawlor et al., 2009; von Jonquieres et al., 2013). Overall AAV2, AAV3 and AAV4 perform comparatively poorly in the CNS with notably reduced overall transduction efficiency and vector spread (Castle et al., 2016).

While direct IP AAV delivery is highly efficient and well tolerated, it requires complex surgeries and in diseases like leukodystrophies that affect large brain areas a trade-off between surgical risk and number of injection-sites is required. Consequently, alternative delivery routes were investigated and following ICV injection, AAV8 and AAV9 were identified as the superior serotypes crossing the CBB, while AAV4 appeared favorable when exclusively targeting the ependymal cell layer (Davidson et al., 2000; Chakrabarty et al., 2013; Bey et al., 2020). Comparison of 12 rAAV vectors for transgene expression in the CNS following IV revealed that particularly AAV7, AAV8, AAV9, AAV.rh8, AAV.rh10, AAV.rh39 and AAV.rh43 crossed the BBB (Gray et al., 2013; Samaranch et al., 2013; Yang et al., 2014). Important advances in natural discovery for CNS applications include novel clade F (AAV9 clade) AAV variants isolated from human CD34^+^ HSC of which AAVHSC7, AAVHSC715 and AAVHSC717 effectively crossed the NHP BBB and transduce astrocytes, oligodendrocytes, neurons and cells of the choroid plexus (Smith et al., 2014; Ellsworth et al., 2019). Since AAVHSCs effectively transduce CD34^+^ HSC they may also be employed in gene editing approaches for HSC-GT (Chatterjee et al., 2020). Using single molecule real time sequencing 81 novel capsids were identified from human tissue interestingly capsid variant AAVv66 crossed the BBB following IV administration and outperformed the prototype AAV2 in CNS biodistribution following both IP delivery (Hsu et al., 2020).

### AAV Capsid Engineering

**FIGURE 1:**
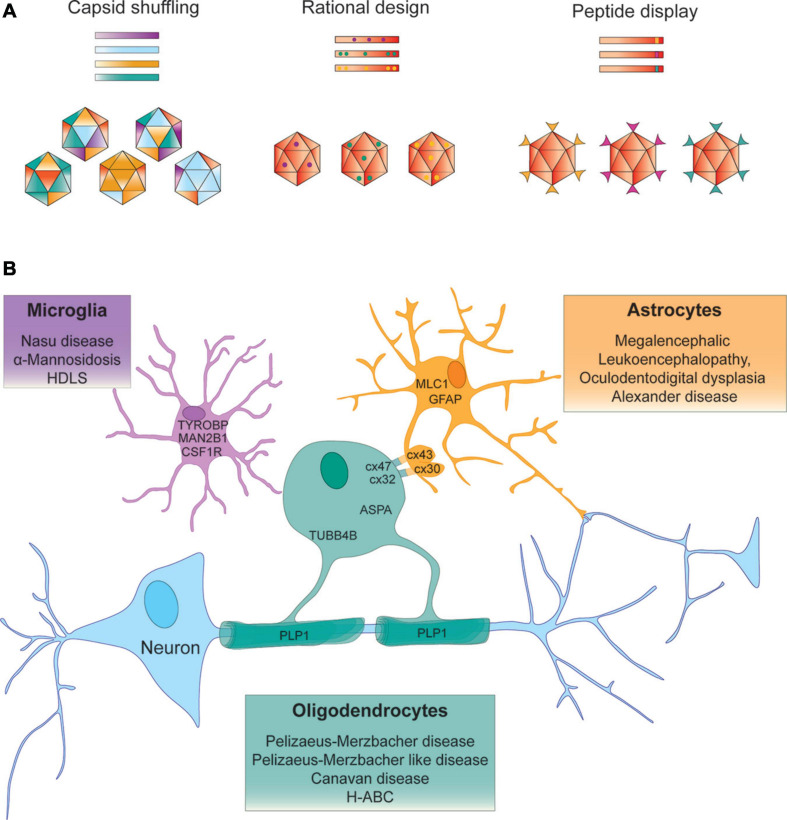
Strategies to identify AAV vectors for AAV – mediated gene targeting to treat leukodystrophies. **(A)** Capsid shuffling uses capsid DNA from different AAV variants to generate chimeric capsid libraries to be screened for desired biological properties. Rational design relies on prior knowledge of structure – function relationships to transfer a predetermined functional aspect to another vector. Peptide display may incorporate peptides from a randomized library for screening purposes or peptides with known function into the AAV capsid. **(B)** Examples of leukodystrophy – specific gene targeting approaches that aim to deliver the therapeutic gene to the neural cell type that expresses the disease-causing gene.

Due to its recognized potential as a gene therapy vector the crystal structure of AAV2, the most abundant naturally occurring AAV in the human population, was resolved in 2002 (Xie et al., 2002). Since then, capsid engineering has evolved rapidly to tailor rAAV vector properties to biomedical needs. Figure 1A depicts common strategies for identifying AAV polymorphisms to improve gene targeting and delivery. Comparison of natural AAV serotypes endemic to humans using high resolution crystallography and electron microscopy indicates that at the surface, the structural diversity of natural AAV serotypes is largely confined to nine variable regions (VR) that enable host interaction including receptor binding, endocytosis, and trafficking. These include depressions at each twofold, marked protrusions surrounding the threefold axis and pores and canyons at each fivefold (Xie et al., 2002; DiMattia et al., 2012; Gurda et al., 2013; Zinn et al., 2015). Five of these VRs are located in prominent protrusions and have been associated with serotype-specific antibody interaction and cell surface receptor binding (Tseng and Agbandje-McKenna, 2014). Transduction is thought to occur through primary proteoglycan receptors mediating attachment while secondary receptors cooperate with entry co-factors to facilitate internalization during AAV uptake. Primary receptors include heparan sulfate (AAV2, AAV3, AAV3b, AAV6), α2,3 and α 2,6N-linked sialic acid (AAV1), α2,3-O-linked sialic acid (AAV4) or terminal N-linked galactose (AAV9), while laminin receptor (AAV2, AAV3, AAV8, AAV9), fibroblast growth factor receptor, αvβ5 integrin (AAV2), and platelet derived growth factor (AAV5) have been identified as secondary receptors (Huang et al., 2014). Notably a pan AAV receptor (AAVR) that is widely distributed across tissues and cell types as well as the G-protein coupled receptor family member 108 (GPR108) have recently been recognized to be essential for efficient transduction of most known human and NHP AAVs (Mizukami et al., 1996; Pillay et al., 2016; Dudek et al., 2020).

To tailor rAAV as a gene therapy vector, important domains of AAV capsids can be harnessed in rational vector design to enhance transduction efficiencies and re-target AAV to select cell types and tissues. Of the five exposed VRs located in the AAV capsid protrusions particularly VR-IV and VR-VIII have been utilized to expand tropism and re-target AAV without interfering with packaging and capsid stability. As this has been covered in reviews elsewhere (Grimm and Büning, 2017; Hüser et al., 2017; Lee et al., 2018; Büning and Srivastava, 2019; Wang et al., 2019; Li and Samulski, 2020) we only give an overview on highlights relevant to the development of CNS targeted gene therapy.

Numerous AAV capsids that have been engineered through capsid shuffling, error prone PCR or scanning mutagenesis and selected for more desirable characteristics, show the highest sequence variability in surface exposed regions (Büning et al., 2015). With potential relevance to the treatment development for some leukodystrophies, a recently identified AAV vector Olig001, generated through capsid shuffling demonstrated > 90% specificity for oligodendrocytes following direct intrastriatal injection into rats and NHPs. The most prominent differences in Olig001 from most parental capsids are located in VR-IV, VR-VII and VR-VIII. Not surprisingly the largest sequence similarity of this capsid is with AAV8, one of the stem vectors that has previously been shown to transduce oligodendrocytes (Powell et al., 2016; Mandel et al., 2017).

Microglia are the most challenging brain cell type to transduce using AAV vectors. Interestingly using targeted mutagenesis, a triple tyrosine mutant of AAV6 that effectively escaped proteasomal degradation targeted gene expression to microglia more effectively. This capsid variant may prove valuable for exploring treatments for select microgliopathies (Rosario et al., 2016; Schober et al., 2016).

More recently, the potential of *in silico* design of AAV capsids was demonstrated by ancestral capsid sequence reconstruction from 75 human and NHP capsid sequences predicting Anc80 as the ancestral node AAVs currently evaluated in clinical trials. Amino acid posterior probability calculation and alignment with AAV2 and AAV8 identified few variable residues and prompted the generation of an Anc80 capsid library, that was subsequently assessed for particle assembly and transduction efficiency *in vitro*. One variant Anc80L65 has since been praised for its outstanding efficiency targeting sensory organs including the inner ear and retina. Anc80L65 has recently been shown to outperform AAV9 targeting the CNS, following IV injection but also revealed elevated transduction in the liver. Anc80L65 predominantly transduced astrocytes and neurons and was found to diffuse more extensively in the CNS following intraparenchymal delivery (Zinn et al., 2015; Hudry et al., 2018; Hudry and Vandenberghe, 2019). In a separate screen of a computationally designed capsid library, *in vivo* selection identified AAV.SCH9 which infected 60% of NPCs and achieved a 12-fold greater transduction volume compared to AAV9 following IP delivery (Ojala et al., 2018).

Efficient AAV uptake is key to successful AAV mediated gene delivery, but transduction efficiency of many capsids show species dependent variation, with the performance of capsids in rodent studies not always translating to larger animal models or humans. Importantly, AAV uptake itself does not guarantee transgene expression. Endosome escape, capsid degradation, intracellular transport, and nuclear translocation are significant hurdles. Only 20% of all AAV2 virions that infected fibroblasts were able to deliver their genetic payload to the nucleus and express the transgene (Hansen et al., 2000). This difference between virion uptake and ‘functional transduction’ resulting in transgene expression is AAV subtype, target cell and species dependent (Lisowski et al., 2014; Westhaus et al., 2020). Capsid phosphorylation at tyrosine and serine/threonine precedes ubiquitination at lysine residues and subsequent proteasomal degradation. Site specific mutagenesis of all surface exposed tyrosine serine, threonine residues and lysine residues has led to the identification of AAV2 mutants that are significantly more resistant to proteasome degradation and increase functional transduction (Zhong et al., 2008; Markusic et al., 2010; Aslanidi et al., 2013; Martino et al., 2013; Li et al., 2015).

Rational capsid design has also enabled intracellular redirection. Through integration of a mitochondrial targeting sequence, the virion directed the AAV expression cassette to mitochondria, where it facilitated transcription of the human NADH dehydrogenase 4 gene in mitochondrial genetic code (Yu et al., 2012).

Aside from optimizing capsids to navigate through the BBB and successfully transduce the target cells, CNS targeting following IV administration of therapeutic AAV requires protecting the capsid from deleterious interaction with the host immune system. Severe adverse events stemming from innate and cellular immune responses have occurred in several clinical trials associated with high-dose systemic AAV gene therapy, the exact mechanisms of which have been subject to recent review (Ronzitti et al., 2020). These include major histocompatibility complex type I (MHCI) mediated presentation of proteolytically processed capsids and the resulting cytotoxic T-cell activation as well as innate immune responses through pattern recognition receptors and the complement system. Due to the large prevalence throughout the population and broad cross-reactivity between serotypes, neutralizing antibodies can significantly counteract gene therapy and consequently, participants in many AAV clinical trials are now routinely screened to rule out pre-existing humoral immunity. More immune-evasive capsid variants including AAV-DJ that retain potent transduction efficiency have been generated through capsid shuffling (Grimm et al., 2008). Similarly, mapping of specific antibody epitopes on the capsids, which also appear enriched in variable regions and particularly in conserved residues along the three-fold axis are subjected to rational design of virions able to evade specific antibodies (Giles et al., 2018). Aside from genetic approaches a number of chemical avenues have been assessed to shield the AAV capsid from the host immune response and other undesired interactions (Kaygisiz and Synatschke, 2020).

### AAV Peptide Display

A different approach to amend AAV tropism is the insertion of peptides into the AAV capsid. Peptides can be inserted into all AAV VPs since encoded in the same open reading frame, targeting VP3 will also alter VP1 and VP2 leading to peptide display on all 60 capsid subunits. Peptide insertions into the AAV2-I587 and I588 sites in VR-VIII destroy the HSPG binding motif and are commonly used in peptide display libraries and screens for cell surface receptor re-targeting (Büning and Srivastava, 2019). Corresponding sites have been used to alter vector tropism in various AAV serotypes and *in vivo* screening of barcoded capsid libraries is rapidly developing. To date only relatively few mutants with select cell type – specific tropism have been published. Notably, AAV9P1 has performed exceptionally well on cultured astrocytes (Kunze et al., 2018; Börner et al., 2020) and AAV.r3.45 with a seven amino acid peptide insertion selected from > 3x10^6^ capsid variants through direct evolution, transduced rat NPCs with great efficiency *in vitro* (Jang et al., 2011). AAV vectors with enhanced retrograde transport efficiencies or select dopaminergic neuron tropism have also been identified, but peptide displaying capsids that selectively transduce oligodendrocytes or microglia remain to be identified (Davidsson et al., 2019).

Significant advances in CNS targeting following IV delivery have been achieved and these are important to reduce the extraordinarily large vector doses needed for IV AAV administration (Yang et al., 2014; Mendell et al., 2017). Due to its ability to cross the BBB, effectively transduce neural cells and its comparatively low prevalence in the human population, AAV9 has emerged as one of the most promising AAV vectors for CNS gene therapy. In a tour de force Adachi et al. used genome barcoding and alanine-scanning mutagenesis combined with biodistribution assays to link AAV9 capsid characteristics including the neutralizing antibody binding epitope and the galactosyl-proteoglycan-binding site to specific amino acids. Subsequent transfer of 10 key amino acids harboring the galactosyl-proteoglycan-binding site into heparin binding deficient AAV2 transferred this tropism characteristic to the resulting AAV vector (Adachi et al., 2014). Separately, identification of the BBB traversing footprint of AAV.rh10 and subsequent grafting of eight amino acids onto AAV1 converted AAV1RX into a BBB traversing vector with reduced transduction in liver and vasculature following IV delivery (Albright et al., 2018). Incorporation of ‘functional transduction’ parameters in the screening of an AAV9 capsid library that contained a randomized seven amino acid peptide in VP3 and coupled the Cap gene with a Cre invertible switch, identified AAV.PhP.B, that crosses the murine BBB ∼ 40-fold more efficiently. Thus biodistribution of AAV.PhP.B was effectively shifted towards the CNS (Deverman et al., 2016). Expansion of the technology has since identified capsid variants that predominantly transduce CNS neurons (AAV.PhP.eB), peripheral neurons (AAV.PhP.S), equally transduce excitatory and inhibitory neurons (AAV.PhP.N) or vascular endothelial cells and astrocytes (AAV.PhP.V1) (Chan et al., 2017; Ravindra Kumar et al., 2020). Functionally, the enhanced BBB traversing efficiency of AAV PHP.B and PHP.eB was linked to glycosylphosphatidylinositol (GPI)-anchored lymphocyte antigen 6 complex (Ly6a) expressed on brain endothelial cells (Hordeaux et al., 2019). Although the improved ability of the above variants to cross the BBB is mouse strain dependent and did not translate to the marmoset, multiplexed Cre-recombination-based AAV targeted evolution (CREATE) identified capsid variant AAV.PhB.C1-3 that demonstrate enhanced efficiency traversing the BBB across all mouse strains and exhibit strong bias towards astrocytes (Hordeaux et al., 2018; Matsuzaki et al., 2018; Ravindra Kumar et al., 2020).

An exciting advance was the targeted insertion of nonviral moieties including designed ankyrin repeat proteins (DARPins) into the AAV capsid to elicit a desired receptor binding function (Büning and Srivastava, 2019). Development of an GluA4 DARPin expressing AAV2 that has a > 90% specificity for interneurons, highlights the potential of this technology (Hartmann et al., 2019). Similarly, AAV re-targeting was achieved via incorporation of unnatural amino acids that enabled peptide or aptamer conjugation to the capsid surface using click chemistry (Kelemen et al., 2016; Katrekar et al., 2018). Notably, switchable AAV2, that was de-targeted from HSPG binding through mutation in the above-mentioned protrusion can bind re-targeting DARPins after vector production (Münch et al., 2015). These studies are framed by the caveat that the human brain is likely to challenge these pre-clinical proof of concept studies, but none-the-less, validate the strategy and translational potential of hybrid AAV vector technologies for leukoencephalopathy-associated gene targeting.

### The Recombinant AAV Genome

The key component of a gene therapy is the genetic payload and the regulation of its expression. The wildtype (wt) ssDNA genome of naturally occurring AAVs spans approximately 4.7 kilobases (kb) and, restricted by virion size, packaging capacity is capped at ∼5 kb or 2.3 kb in self-complementary (sc) genomes (Dong et al., 1996). scAAV genomes are palindromic and form a double stranded DNA in the transduced cell to facilitate rapid transgene expression without prior DNA replication (McCarty, 2008). With the exception of the terminal inverted repeats (ITRs), the only *cis*-acting element (CRE) required for rAAV vector production, the entire wt genome is replaced by the recombinant expression cassette consisting of promoter, genetic payload and regulatory elements. The ITRs harbor the essential replication and packaging signals and are vital for episome formation and protection from cellular nucleases. Most AAV vectors are produced via cross-packaging where ITRs and Rep genes from one serotype are packaged into the capsid from another. The most commonly used ITRs stem from AAV2 (Wilmott et al., 2019).

Ideally a gene therapy should aim to restore as close to natural gene expression profile as possible to reset the proteomic and metabolomic micro-environment. Genetic payloads and mechanisms to control transgene expression have become increasingly versatile and can include complete coding sequences for proteins, untranslated regions (UTRs) and regulatory elements beyond the polyadenylation signals including post transcriptional regulatory elements, ribozymes, aptamers, regulatory RNAs like short hairpin RNAs (shRNAs), microRNAs (miRNAs), other non-coding RNAs and guide RNAs for gene editing (Domenger and Grimm, 2019; Wang et al., 2020). These combined aspects of the AAV toolkit need to be considered in a gene therapy strategy for gene replacement, gene editing, gene addition and to modulate or abrogate target gene expression. Ubiquitous unregulated expression of a genetic payload can have detrimental side effects. Consequently, implementation of mechanisms that restrict and control transgene expression are rapidly gaining attention in the field.

The first level of gene regulation is governed by the promoter that recruits transcription factors (TFs) in a sequence specific manner to initiate transcription through RNA polymerases. The proximal core promoter is generally located within the first 1 kb of the transcription start site, but additional *cis*-regulatory elements (CREs) carrying clusters of binding sites for silencers and enhancers may be located up to 1 megabase pair up or downstream in intergenic or intronic regions. rAAV – mediated transgene expression has predominantly employed strong constitutive eukaryotic, viral or hybrid promoters that are thought to drive expression in all cell types. Prominent examples include the cytomegalovirus (CMV) promoter, the phosphoglycerate kinase (PGK), elongation factor 1 α (EF1α), ubiquitin C (UbiC) and chicken β-actin promoter and various hybrids thereof. The 1.6kb CAG promoter comprises the CMV immediate early enhancer and the chicken β-actin promoter fused to the first exon and intron and the splice acceptor of the rabbit β-globin gene. In light of the limited packaging capacity of AAV vectors, size reduction of the of CAG promoter hybrids to ∼ 800 bp by replacing the 5’-UTR of CBA with either a truncated simian virus 40 intron or minute virus of mice VP intron were welcomed achievements (Wang et al., 2003; Gray et al., 2011). CBA hybrids with the CMV immediate early enhancer (CAGGS) a truncation thereof (CBh) enable long term transgene expression in the CNS. CMV and CBA promoter hybrids are now employed in FDA approved AAV drugs (Niwa et al., 1991; Klein et al., 2002; Gaudet et al., 2013; Mendell et al., 2015, 2017; Armbruster et al., 2016; Weleber et al., 2016; Pennesi et al., 2018; Al-Zaidy et al., 2019; Gray et al., 2019). Direct intraparenchymal injections of different AAV serotypes into the mammalian CNS employing these constitutive promoters yielded strong, predominantly neuronal transgene expression, thus giving AAV the reputation to be a largely neurotropic vector, but most AAV serotypes and variants have since been shown to have a relatively broad tropism.

While capsid evolution and rationally designed AAV variants may home in on specific cell types, employing cell type – specific promoters can restrict transgene expression to select cell types (Klein et al., 2008; Lawlor et al., 2009; von Jonquieres et al., 2013; Gessler et al., 2019). Among the first, the relatively large 1.8 kb human neuron specific enolase (NSE) promoter, the 1.5 kb human platelet derived growth factor (PDGF) promoter and the 1.3 kb Ca^2+^/calmodulin kinase II (CamKII) promoter successfully to drive AAV – mediated transgene expression in neurons. Shorter neuron specific promoters including the 0.47 kb human synapsin (hSYN1) promoter, a compacted 0.4 kb truncated neuronal-specific CamKII promoter and the 0.23 kb methyl CpG binding protein (MeCP2) promoter have been generated since (Peel et al., 1997; Kügler et al., 2001; Shevtsova et al., 2005; Nathanson et al., 2009; Gray et al., 2011; Chandler et al., 2017).

With regards to AAV gene therapies targeting some leukodystrophies, promoter mediated restriction of transgene expression to glial cells is desirable. The 2.2 kb human glial acidic glycoprotein (GFAP) promoter was the first to show clear astrocyte specificity in the AAV setting (Brenner et al., 1994). Following identification of a *cis*-regulatory element (CRE) required for silencing activity in neurons, this promoter has since been compacted to a twofold more active 681 bp gfaABC_1_D promoter with comparable astrocyte specificity (Lee et al., 2008) and a gfa2(B_3_) variant targeting GFAP positive astrocytes in mouse basal ganglia. More recently, following the identification of aldehyde dehydrogenase 1 like protein 1 (ALDH1L1) as a pan-astrocytic marker, the 1.3 kb human ALDH1L1 promoter has restricted AAV – mediated transgene expression to astrocytes in certain brain regions in mice (Cahoy et al., 2008; Koh et al., 2017).

Oligodendrocyte specific expression in the context of AAV was first achieved with the 1.9 kb mouse myelin basic protein promoter (Mbp) promoter, but heavily depends on the timepoint of AAV infusion after onset of myelination in mice (Chen et al., 1999; Lawlor et al., 2009; von Jonquieres et al., 2013). This promoter has since been used to target oligodendrocytes in preclinical gene therapies for Pelizaeus – Merzbacher like disease and for CD (Georgiou et al., 2017; von Jonquieres et al., 2018). Following identification of the myelin gene regulatory factor (MRF) as a critical transcriptions factor regulating myelination in the CNS and characterization of its consensus binding motif (Emery et al., 2009; Koenning et al., 2012), screening for this motif in evolutionary conserved regions in close proximity to the transcription start sites of known myelin associated genes, identified the 0.3 kb human myelin acidic glycoprotein (MAG) which was able to restrict AAV – mediated expression to oligodendrocytes *in vivo* (von Jonquieres et al., 2016). More recently oligodendrocyte specific expression of a synthetic miRNA was achieved using the 1.8 kb human 2’,3’-cyclic nucleotide 3- phosphodiesterase (CNP) proximal promoter (Li et al., 2019).

In the context of targeting microglia, several promoters including CD68 and CD11b have been assessed with the F4/80 promoter being the most promising to date (Rosario et al., 2016). However, CD68 is upregulated in activated phagocytosing microglia and consequently the CD68 promoter may prove more effective and valuable in a disease setting (Zotova et al., 2013).

Founded on the growing availability of genomic data new bioinformatic strategies for rational and synthetic promoter design are evolving. Transcription is governed through large and convoluted deoxyribonucleoprotein complexes that can often harbor both repressor and activator potential. Promoter activity depends on the tissue, cell type and environment specific nucleoproteome, chromatin condensation and epigenetic footprint within the given cell. Higher throughput bioinformatic directed parallel design for example through the Pleiades Promoter Project has identified cell type and tissue specific core promoter regions and CREs. Subsequent compaction of the core promoter and multiple conserved computationally – predicted CREs to AAV compatible ‘MiniPromoters’ has led to the identification of four glial specific promoters stemming from S100B (Ple266), UTG8 (Ple267) and Olig1 (Ple304, Ple305) (Portales-Casamar et al., 2010; de Leeuw et al., 2016). Many CREs have been shown to retain their activity when used as building blocks in modular synthetic or compacted promoters (Tornøe et al., 2002; Lee et al., 2008; Domenger and Grimm, 2019). Screening of a barcoded library containing 230 synthetic promoters incorporating similar bioinformatic designs found 11% of promoters drive expression in specific cell types in the CNS. Notable examples are ProC17 which efficiently restricts AAV PhP.B mediated transgene expression following IV injection to parvalbumin positive neurons in the CNS, while ProB12 generated by ordered assembly of evolutionary conserved CREs directed transgene expression to a subpopulation of protoplasmic astrocytes (Jüttner et al., 2019). With regards to promoter development in the AAV context, it is important to note, that the ITRs themselves have weak inherent promoter activity and CRE have been identified in AAV2 ITRs that may affect the transgene expression (Flotte et al., 1992, 1993; Haberman et al., 2000; Logan et al., 2017). In addition, it has recently been observed, that promoter – capsid interactions cause a shift in the AAV9 gene expression profile from neurons to oligodendrocytes demonstrating that interactions with the capsid and the recombinant genome may affect functional transduction in a cell type – specific manner *in vivo* (Powell et al., 2020). This fundamental discovery emphasizes the importance of screening capsid and expression cassette elements for functional transduction in conjunction (Bohlen et al., 2020; Powell et al., 2020). Together these findings underscore the importance of integrating screening for functional transduction in humanized animal models replicating target cell and disease state as closely as possible.

In the case of liver directed gene therapies, this was achieved by repopulating the liver of *Fah^–/–^; Rag2^–/–;^ Il2rg^–/–^* (FRG) mice with primary human hepatocytes where screening for functional transduction revealed that bioengineered capsids outperform natural serotypes (Lisowski et al., 2014; Westhaus et al., 2020). While to our knowledge this has not yet been done in the brain, appropriate models are available. For example, the CNS of the *Mbp^–/–^ Rag2^–/–^* shiverer mouse has successfully been repopulated with human glial progenitors and human iPSC (Wang et al., 2013; Windrem et al., 2014; Osorio and Goldman, 2016). Similarly, the recent advances in preclinical cell therapy for CD using human iPSCs may lend itself to similar screens in the CNS (Feng et al., 2020). In fact, AAV – mediated expression of Oct4, Klf, Sox2, and Myc has also been used to induce pluripotency (Senís et al., 2018).

Ideally a therapeutic transgene should self – regulate its own expression tailored to the requirements of the disease state. This might also be achieved by CREs in the promoter responding to metabolic or physiological changes associated with specific disease states in future. An exemplar is the incorporation of hypoxia response elements into the astrocyte specific, compacted GfaABC_1_D variant generating a hypoxia – inducible GFAP promoter (Prentice et al., 2011). Similarly, introduction of a ARF5/AuxRE transcription factor/response element enabled cleaved caspase 3 dependent transcriptional activation in cells undergoing apoptosis (Vagner et al., 2015). It remains to be determined, if similar system enable autoregulation of therapeutic gene expression controlled by the disease state in leukodystrophies.

A different advance was the incorporation of riboswitches that facilitate inducible inhibition of a highly active synthetic ribozyme that reliably cleaves the transgenic mRNA unless inhibited with a specific antisense oligonucleotide or small molecules, thereby effectively allowing control of transgene expression (Strobel et al., 2020; Zhong et al., 2020). Another key gene therapy safety element that is under investigation is the control of AAV – mediated transgene expression in off-target tissue where transgene expression may be undesirable or induce an immune response. Because these strategies have been recently reviewed, we will only highlight a notable advance with direct relevance to CNS targeted gene therapy (Domenger and Grimm, 2019; Ingusci et al., 2019).

RNA interference, through incorporation of miRNAs can either be driven by cell type – specific RNA polymerase lI promoters and/or incorporated in the 3’UTR of the transgenic mRNAs. The latter is possible because the active secondary structure of many miRNAs is determined by specific RNA binding proteins and the alternative splicing machinery differs among cell types. Notably, a successful IV AAV9-*ASPA* gene replacement therapy in a CD mouse model, incorporated a miRNA with muscle specific miRNA-1 and liver specific miRNA-122 sites ablating transgenic *ASPA* expression in these organs, but not in the CNS (Ahmed et al., 2013).

Particularly in the case of gene replacement in which the host’s immune system is naïve to the transgene, transduction of antigen-presenting dendritic cells, macrophages or B-lymphocytes can lead to transgene – derived peptide presentation on MHCI molecules and trigger CD8^+^ cytotoxic T-cell activation. Incorporation of the hematopoietic lineage specific synthetic miRNA-142 targeting the transgene in the 3’UTR of the same AAV vector was sufficient to suppress this immunotoxicity to the point that allowed re-administration of the same transgene albeit with a different AAV serotype (Xiao et al., 2019). This strategy may also mitigate immune reactions targeting the bacterial Cas9 transgene in AAV – mediated CRISPR gene editing *in vivo*. Strategies and advances in this field can be pursued via a recent review (Wang et al., 2020).

## Considerations for the Next Generation of Gene Therapies for Leukodystrophies

Although currently assessed in clinical trials, rAAV vector spread may be a limiting factor in intracerebral treatment of neurological diseases affecting large areas of the CNS (Hudry and Vandenberghe, 2019). In fact, it has been estimated, that over 100 needle tracts would be required to achieve adequate coverage of the entire human brain (Hinderer et al., 2018a). Since the discovery of BBB and CBB traversing AAV vectors including AAV9 and growing confidence in their excellent safety profile, the prospect of achieving widespread gene transfer to the CNS via non-invasive vector delivery attracted great attention. Following a breadth of supportive clinical trials this advanced to regulatory approval of Zolgensma^®^. However, the recent tragic deaths following severe hepatobiliary disease, bacterial infection and sepsis associated with high dose (3 x 10^14^ vg/kg) IV delivery of AAV vector (AAV8-MTM1) in the ASPIRO phase I/II clinical trial targeting X-linked myotubular myopathy (NCT03199469), is a reminder that despite comprehensive pre-clinical safety and efficacy studies and the excellent safety profile of AAV even at high doses, outcomes in first human trials may result in the severest adverse events (Philippidis, 2020; Wilson and Flotte, 2020). Growing safety concerns regarding liver toxicity in nonhuman primates and pigs subjected to systemic high dose (AAV9-SMN, 1.2x10^14^ vg/kg) and genotoxic integration potentially increasing the risk of liver cancer founded on clonal hepatocyte expansion observed in dogs (AAV9-cFVIII, 2.5x10^13^ vg/kg) emphasize the importance of continued pursuit of the safest and most efficient AAV vectors, particularly for the treatment of leukodystrophies in which the entire CNS needs to be targeted, which inherently requires a high vector dose (Hinderer et al., 2018b; Nguyen et al., 2021). This entails AAV vector development with enhanced ability to traverse the BBB and/or CBB. In addition, de-targeting the therapeutic rAAV capsid from off-target organs and improving transduction of a selected cell types and tailored therapeutic gene expression to these cells will reduce the vector dose required to achieve therapeutic benefit. At present, regardless of the disease, experience in clinical trials with the current gold standard AAV9 indicates effective CNS targeting via IV, IT or ICM delivery requires vector doses above 1x10^14^ vg/kg. However, as discussed above promising progress is made in vector development and up to 40-fold dose reductions are already achievable in mice (Deverman et al., 2016, 2018). In addition, it is increasingly evident that gene therapy for many genetic diseases would benefit from a cell type targeted and inducible approach, that restricts transduction and/or expression of the genetic payload to a specific target cell type. Table 1 gives an overview of naturally – occurring and engineered AAV capsids and promoters observed when expressing green fluorescent protein and similar fluorescent markers, that may be considered when targeting neural cell types. Ongoing development in this area of the next generation gene therapies will be relevant to the treatment of select leukodystrophies and leukoencephalopathies. Gene therapy should first and foremost be safe, halt disease progression, improve the quality of life by resolving the underlying pathophysiological cause and ultimately aim to restore as close to natural gene expression profile in the CNS while minimizing potential off target effects associated with the treatment. With this focus in mind, Table 2 summarizes the publicly available cellular expression profile of known leukodystrophy associated genes, founded on RNA sequencing of neural cells isolated from the murine cerebral cortex (Zhang et al., 2014). Inclusion of leukodystrophies in Table 2 is founded on a classification according to pathogenic mechanisms and pathological changes (van der Knaap and Bugiani, 2017). The leukodystrophies selected are exemplary and include leukodystrophies from each category with a clear CNS white matter phenotype, however this selection is not meant to be exhaustive. Figure 1 provides a graphical overview of targeting strategies for leukodystrophy gene therapy development when aiming to restore the normal, natural cell type specificity of gene expression.

**TABLE 1:** Summery of AAV capsids and cell type targeted promoters that achieve AAV mediated transgene expression in neural cells.

	Capsid*	*Promoter*
*Neurons*	**AAV1, AAV9, AAV.rh10, AAV.v66, Anc80L65, PhP.B, PhP.eB,** AAV2, AAV5, AAV7, AAV8, AAV.rh8, AAV.rh20, AAV.rh39, AAV.cy5 AAVHSC7,15,17, AAV.PhB.C1-3 6	*NSE* *PDGF* *CamKII* *hSYN1* *MeCP2* *ProC17* *Ple155*
*Astrocytes*	**AAV8, AAV.rh43, Anc80L65, AAV.PhB.C1-3, AAVHSC.7, AAVHSC.15, AAVHSC.17,** AAV1, AAV2, AAV5, AAV7, AAV8, AAV9, AAV.rh8, AAV.rh10, AAV.rh20, AAV.rh39, AAV.cy5, AAV.v66	*GFAP* *gfaABC1D* *gfa2 (B_3_)* *ProB12* *Ple266* *Ple267*
*Oligodendrocytes*	**AAV8, Olig001, AAV.rh10 AAV9**, **AAV.rh20, AAV.rh39, AAV.cy5,** PhP.B, AAV.v66, AAVHSC7,15,17, AAV1/2	*Mbp* *MAG* *CNP* *Ple304* *Ple305*
*Microglia*	**rAAV6, AAV.v66,** AAV5, AAV7, AAV8, AAV9, AAV.rh10	*F4/80* *CD68* *CD11b*
*Ependymal cells & vascular endothelia*	** PhP.V1**, AAV1, AAV4, AAV5, AAV7, AAV8, AAV9	*Ple261*
*Neural progenitor cells*	**AAV.SCH9, AAV.r3.45**	to be identified

*Bold font indicates superior transduction was reported for the AAV capsid.

**TABLE 2:** Gene expression of leukodystrophy associated genes across neural cell types.

Disease	Gene	Genebank ID	Astrocyte	Neuron	OPCs	Oligo-dendrocyte	Microglia	Vascular endothelial cells
**Hypomyelinating**								
Pelizaeus-Merzbacher like disease	*Gjc2*	*118454*	-	-	-	+++	-	-
Pelizaeus-Merzbacher disease	*Plp1*	*18823*	-	-	-	+++	-	-
Hypomyelination with atrophy of the Basal Ganglia and cerebellum (H-ABC)	*Tubb4a*	*22153*	-	-	-	+++	-	-
Sox10 associated PCWH syndrome	*Sox10*	*20665*	-	-	++	++	-	-
**Vacuolating**								
Canavan disease	*Aspa*	*11484*	-	-	-	+++	-	-
Cx32- related Charcot-Marie-Tooth disease	*Gjb1*	*14618*	-	-	-	+++	-	-
**Demyelinating**								
Krabbe disease	*GalC*	*14420*	+	-	++	++	-	+
Metachromatic leukodystrophy	*Arsa*	*11883*	+	+	+	+	+	+
X-linked adrenoleukodystrophy,	*Abcd1*	*11666*	-	-	-	+	++	-
Multiple sulfatase deficiency	*Sumf1*	*58911*	+	+	+	+	++	+
**Astrocytopathies**								
Megalencephalic leukodystrophy with subcortical cysts	*Mlc1*	*170790*	+++	-	-	-	-	-
Oculodentodigital Dysplasia with cerebral white matter abnormalities	*Gja1*	*14609*	+++	-	-	-	-	+
Alexander disease	*Gfap*	*14580*	+++	-	-	-	-	-
CIC2 related leukoencephalopathy	*Clcn2*	*12724*	++	+	+	++	-	-
Vanishing White Matter Disease	eIf2b1 – 5*	*209354, 217715, 108067, 13667, 224045*	+	+	+	+	+	+
Aicardi Goutieres Syndrome	*Rnaseh2a, b, c*	*69724, 67153, 68209*	+/+/+	+/+/+	+/++/+	+/+/+	+/+/++	+/+/++
**Microgliopathies**								
Hereditary diffuse leukoencephalopathy with axonal spheroids (HDLS)	*Csf1R*	*12978*	-	-	-	-	+++	-
Nasu disease	*Tyrobp*	*22177*	-	-	-	-	+++	-
α-Mannosidosis	*Man2b1*	*17159*	-	-	-	-	+++	-

**Disease**	**Gene**	**Genebank ID**	**Astrocyte**	**Neuron**	**OPCs**	**Oligo-dendrocyte**	**Microglia**	**Vascular endothelial cells**

**Leuko-axonopathies**								
Developmental and epileptic encephalopathy 29	*Aars1*	*234734*	+	+	+	+	+	-
Leukoencephalopathy, progressive with ovarian failure	*Aars2*	*224805*	+	+	+	+	+	+
Hypomyelination with brainstem spinal cord involvement & leg spasticity	*Dars1*	*226414*	+	+	+	+	-	+
Leukoencephalopathy w brainstem, spinal cord involvement, elevated lactate	*Dars2*	*226539*	+	+	+	+	+	+
Combined oxidative phosphorylation deficiency 12	*Ears2*	*67417*	+	+	+	+	+	-
Leukodystrophy, hypomyelinating 9	*Rars1*	*104458*	+	+	+	+	+	-
AIMP1 related disease	*Aimp1*	*13722*	-	+	+	+	-	+
H4 leukodystrophy	Polr3a, b*	*218832, 70428*	+	+	+	+	+	+
GM1 Gangliosidosis	*Glb1*	*12091*	+	+	+	+	+	-
Tay-Sachs GM2 Gangliosidosis	Hexa, Hexb*	*15211*	-	-	-	-	+++	-
Sanfilippo syndrome (MPS IIIA)	*Naglu/Sgsh*	*27419, 27029*	- / +	- / -	- / +	- / +	++	- / +
**Leuko-vasculopathies**								
Cerebral autosomal dominant arteriopathy with subcortical infarcts and leukoencephalopathy (CADASIL)	*Notch3*	*18131*	+++	-	-	-	-	+
Cerebral autosomal recessive arteriopathy with subcortical infarcts and leukoencephalopathy (CARASIL)	*Htra1*	*56213*	++	-	+	-	-	+

*Expression pattern of genes in this family are similar.

## Leukodystrophies That May Benefit From Oligodendrocyte Targeting

Oligodendrocytes are the myelin forming cell in the CNS, where they provide axon insulation and enable saltatory impulse propagation which is critical to ordered connectivity between brain regions and with the peripheral nervous system (PNS), where this role is taken by Schwann cells (Kuhn et al., 2019). Particularly during active myelination oligodendrocytes have among the highest metabolic rates in any cell type and require high levels of iron, an essential cofactor in the mitochondrial respiratory chain. In combination with their high turnover, the elevated production of reactive oxygen species and low level of glutathione make oligodendrocytes particularly vulnerable to oxidative stress and free radical formation culminating in lipid peroxidation and cell death (Bradl and Lassmann, 2010). This explains why white matter and oligodendrocyte pathology is prevalent in many leukodystrophies where the mutated gene is not primarily expressed in oligodendrocytes. Nevertheless, RNA sequencing data summarized in Table 2 indicates that in many hypomyelinating and some vacuolating leukodystrophies the mutated gene is almost exclusively expressed in oligodendrocytes, while in many demyelinating leukodystrophies the expression pattern is often broader.

### Pelizaeus-Merzbacher Disease

Pelizaeus-Merzbacher disease (PMD) is a X-linked, recessive dysmyelinating leukodystrophy with the most common and severe forms caused by proteolipid protein 1 (*PLP1*) gene duplication (Inoue et al., 1996; Hudson, 2003; Osório and Goldman, 2018). Resulting PLP1 overexpression triggers oligodendrocyte dysfunction and prevents proper myelin formation leading to classic PMD. This presents with nystagmus, head tremor, systemic hypotonia and hypomyelination usually in the first year of life, followed by spasticity and progressive motor and cognitive decline (Osório and Goldman, 2018). Notably, without addressing the clinical cause, a pre-clinical trial found feeding PMD mice a high cholesterol diet delayed PMD pathology and preserved myelin (Saher et al., 2012). Whether the high dose cholesterol diet is beneficial or sustainable in humans remains to be determined.

Addressing the causative overexpression of *PLP1* was recently achieved using oligodendrocyte specific RNA interference in PMD mice. To restore as close to natural PLP1 protein levels as possible, careful choice of the miRNA backbone, targeting sequence and promoter is needed to avoid off-target effects and RNA interference (RNAi) mediated toxicity. Direct intraparenchymal injection of scAAV1/2 mediating oligodendrocyte restricted expression of a synthetic miR-155 harboring a PLP1 directed short hairpin prevented oligodendrocyte demise, restored myelin, and improved neurological phenotypes and survival (Li et al., 2019). These findings indicate that AAV – mediated oligodendrocyte targeted RNAi for PMD holds promise when the disease is caused by gene duplication.

In contrast to CNS myelin formed by oligodendrocytes, peripheral myelin generated by Schwann cells can tolerate gene duplication but is more vulnerable to nonsense mutations in the PLP1 gene (Shy et al., 2003). Thus, when targeting gene duplication derived PMD, ICM AAV delivery (found to be the superior CSF delivery route in non-human primates) may be the most promising route of administration, mitigating the risk of complex surgery associated with direct multisite intraparenchymal injection and of immune – response and high off target transduction from intravenous delivery (Hinderer et al., 2014). This relies on a capsid that efficiently crosses the CBB via transcytosis of the ependymal layer and readily transduces oligodendroglial cells in the brain. This is currently achieved with AAV9 (Hinderer et al., 2018a). However, more efficient capsid variants are likely to become available enabling dose reduction.

PLP1 is the prototype of a dosage sensitive gene with missense mutations leading to absence of PLP1 causing much milder disease while PLP1 nonsense mutations generating misfolded or incomplete PLP1 frequently promote dysmyelination and early oligodendrocyte death. In these cases, gene replacement in combination with RNAi is unlikely to achieve the exact non-toxic gene dose required during oligodendrocyte differentiation, myelination, and maintenance. Therefore, a gene editing approach may be a better gene therapy option, but immunological complications associated with bacterial Cas9 expressions will still need to be resolved. Antisense oligonucleotides (ASOs), particularly when packaged into oligodendrogliotrophic exosomes that effectively cross the BBB are another developing technology that may hold promise in future. Indeed, therapeutic promise has recently been demonstrated in the ‘jimpy’ mouse model of PMD where single administration of a *Plp1* – targeting ASO restored oligodendrocyte numbers, increased myelination, improved motor performance and extended lifespan (Elitt et al., 2020).

### Pelizaeus-Merzbacher Like Disease

Pelizaeus-Merzbacher like disease (PMLD) is a hypomyelinating leukodystrophy with very similar clinical manifestation to PMD but caused by autosomal recessive loss-of-function mutations in the gap junction protein connexin 47 (*GJC2*) gene (Orthmann-Murphy et al., 2007). High levels of GJC2 are found exclusively in the CNS where it is selectively expressed by oligodendrocytes (Table 2). AAV1/2 – mediated *Mbp* promoter – driven *GJC2* gene replacement by IP injection re-established oligodendrocyte gap junction connectivity and rescued the severe demyelination in a mouse model of PMLD. This underscores the therapeutic potential of an oligodendrocyte targeted AAV – mediated gene therapy (Georgiou et al., 2017). Given the high *GJC2* expression throughout the CNS white matter including in the spinal cord, broad CNS targeting is required and likely sufficient and achievable through ICM delivery into the CSF which can reduce the vector doses and potential immunogenicity, toxicity, and off target risks of systemic IV administration (Nualart-Marti et al., 2013; Hinderer et al., 2014, 2018a,b).

### Hypomyelination With Atrophy of the Basal Ganglia and Cerebellum

Typically, H-ABC presents in early childhood with varied rate of progression and severity involving complex pathology including spasticity, dystonia, dyskinesia ataxia and tremor. Motor function is commonly more severely affected than cognition. H-ABC has been associated with toxic ‘gain of function’ mutations in the TUBB4a gene encoding the microtubule associated protein tubulin β-4a (Nahhas et al., 1993; Hamilton et al., 2014; Curiel et al., 2017). Resolution of the cell type – specific expression in the mouse brain reveals that TUBB4a is almost exclusively expressed in the oligodendroglial lineage in the cortex, while in humans low expression in astrocytes and neurons was detected (Zhang et al., 2014, 2016). Recent development of an accurate mouse model of H-ABC now opens up pre-clinical investigation (Sase et al., 2020). Based on the gain of function hypothesis, a potential therapy would be largely oligodendrocyte focused, directed to reduce mutated tubulin β-4a protein while restoring its wt form. This may be achieved via AAV – mediated expression of a synthetic *TUBB4a* miRNA. To prevent potential undesired effects associated with loss TUBB4A, targeting the miRNA to the untranslated region and simultaneous expression of transgenic wt *TUBB4a* both driven by a predominantly oligodendrocyte – specific promoter may be viable. The AAV serotype chosen, and associated delivery route, would need to efficiently transduce oligodendrocytes. Similar to the above, AAV – CRISPR mediated gene correction and potentially ASO may become additional treatment options in future.

### SOX10-Associated PCWH

SOX10-associated peripheral demyelinating neuropathy, central dysmyelinating leukodystrophy, Waardenburg syndrome, and Hirschsprung disease (PCWH) presents with a complex central and peripheral pathology (Sánchez-Mejías et al., 2010). Because Sox10 is a transcription factor known to drive oligodendrocyte differentiation and myelination, Sox10-associated PCWH would rely on *SOX10* gene replacement in oligodendroglial progenitor cells (OPC). The infantile forms may be particularly amendable to AAV – mediated gene replacement, but suitable capsids enabling selective OPC transduction following efficient delivery to the CNS across the CBB or BBB and appropriate promoters remain to be identified. Potentially AAV variants SCH9 or AAV.r3.45 that have been shown to effectively transduce NPCs are promising candidates.

### Canavan Disease

Canavan disease is a spongiform leukodystrophy caused by loss-of function mutations in the oligodendroglia aspartoacetylase (*ASPA*) encoding gene. Its substrate *N*-acetylaspartate (NAA) is the second most abundant brain metabolite, being predominantly synthesized by aspartate-*N*-acetyltransferase NAT8L in neuronal mitochondria. Although the exact function of NAA remains enigmatic, lack of NAA hydrolysis by ASPA which is exclusively expressed in oligodendrocytes causes its toxic accumulation and severe white matter vacuolization. Age of onset generally correlates with disease severity and in the most common infantile form children present with hypotonia, macrocephaly and fail to reach developmental milestones in months after birth before progressively losing motor function, seizure development and premature demise (Hoshino and Kubota, 2014; Gessler and Gao, 2016). CD was the first CNS disease to ever be treated in a gene therapy in humans more than 20 years ago, utilizing a lipid-entrapped, polycation-condensed delivery system (LPD) with an *ASPA* encoding plasmid DNA (Leone et al., 2000). Later, pre-clinical AAV – mediated *ASPA* gene replacement benefit was observed following intraparenchymal injection of a first generation rAAV2-CBA-*ASPA* vector into CD mice and rats (Matalon et al., 2003; McPhee et al., 2005). Since that time due to the availability of accurate rodent models (Matalon et al., 2000; Traka et al., 2008; Ahmed et al., 2013; von Jonquieres et al., 2014), several pre-clinical and clinical studies have achieved promising outcomes using AAV – mediated gene replacement, RNA interference and recently cell therapy advancing understanding and prospect of the field. In 2013 the Gao lab was first to employ IV rAAV-CAG-*ASPA* delivery using AAV9, AAV.rh8 and AAV.rh10 vectors and included a liver, heart and skeletal muscle de-targeting miRNA. In a CD mouse model, injections up to postnatal day (P)20 targeted predominantly neurons and achieved long-lasting therapeutic benefit by lowering NAA levels, preventing spongiform white matter vacuolization and markedly improved motor performance (Ahmed et al., 2013). This outcome was further improved in the next generation vectors targeting astroglia with codon optimized *ASPA.* Intravenous AAV9-phGFAP-*ASPA* at P1 achieved widespread ASPA expression throughout the CNS and completely reversed the CD phenotype to the point that at the highest dose treated animals performed better than their untreated WT littermates in some behavioral tests (Gessler et al., 2017). In a separate study, targeting oligodendrocytes via direct intraparenchymal injection of AAV.cy5-Mbp-*ASPA* at P30 following disease onset, achieved near complete correction of the CD phenotype including NAA levels vacuolization and motor performance (von Jonquieres et al., 2018). Following the realization that NAA is largely dispensable, at least for myelination and motor function in mice (Guo et al., 2015; Maier et al., 2015; von Jonquieres et al., 2018), an shRNA mediated *Nat8l* knockdown strategy using P1 ICV and ICM delivery of AAV8-U6-*Nat8l*-shRNA achieved comparable therapeutic outcome indicating that in the absence of oligodendroglial ASPA reduction of NAA levels provides key therapeutic benefit (Bannerman et al., 2018). Phenotypic improvement was achieved regardless of the target cell, strategy or delivery route as long as widespread ASPA expression was achieved throughout the CNS. This is underscored by the observation that induced pluripotent stem cells directly injected into the CD mouse CNS differentiated into astrocytes, oligodendrocytes and neurons providing therapeutic benefit comparable to gene therapy approaches (Feng et al., 2020).

Pioneering AAV – mediated gene therapy for leukodystrophies in humans, direct intraparenchymal injection of AAV2-*ASPA* into six sites was performed in phase I clinical trials. Importantly, emphasizing safety and underscoring the immune privilege of the CNS, no neutralizing antibodies and only mild immune response was detected (McPhee et al., 2006). In a 10-year follow up study modest improvement of NAA levels and seizure frequency were accompanied by slowed atrophy and stabilization of clinical status (Leone et al., 2012). Looking forward, glial cell targeted *ASPA* replacement employing novel capsids in combination with peripheral organ de-targeting should enable dose reduction for CSF or IV administration and holds significant promise for CD gene therapy. In addition, NAT8L targeted ASOs were able to promote rapid NAA reduction and reduce vacuolization following postsymptomatic intracisternal administration (Hull et al., 2020). It will be exciting to validate the benefit of presymptomatic and/or repeated ASO administration particularly in the early stage of the disease stage when combined with *ASPA* gene replacement, as *Nat8l* targeting ASOs rapidly decrease NAA load bridging valuable time until transgenic ASPA expression is fully established. Based on the natural ASPA expression pattern, identification of capsid and promoter combinations that efficiently target AAV mediated ASPA expression to human glia will likely hold clinical promise. Parental capsids included in functional transduction screens for efficient human oligodendrocyte specific vectors that cross the BBB and CBB should include Olig001, that has been reported to selectively transduce oligodendrocytes in mice (Powell et al., 2016, 2020).

### Krabbe Disease (Globoid Cell Leukodystrophy)

Krabbe disease is a demyelinating leukodystrophy caused by autosomal recessive mutations of the galactosylceramidase (*GALC*) gene rendering the lysosomal hydrolytic enzyme that is required for myelin turnover inactive. This leads to toxic accumulation of psychosine, a byproduct of galactosylceramide synthesis and GALC substrate (Orsini et al., 1993; Weinstock et al., 2020). In the frequent severe form, children present with hypersensitivity, spasticity, arrested psychomotor development often rapidly deteriorating with seizures leading to death before 3 years of age (Duffner et al., 2011). Currently, pre-symptomatic bone marrow transplant (BMT) and HSCT aiming to restore some level of GALC in the CNS through microglia, are the only treatment options but even these only slow disease progressions.

Based on the severity of the disease, therapeutic approaches spanning from substrate reduction and enzyme replacement to cell- and AAV- mediated gene therapies using a number of different capsids (AAV1, 2, 5, 9, rh.10 and Olig001) and delivery routes (IP, IV, IT, ICM, ICV) have been trialed in mouse models of the disease, many lagging behind expectations. The severity of the disease and extent of gene replacement required to treat Krabbe disease is underscored by the fact that even in combination with bone marrow transplant it required neonatal IV, IT and intraparenchymal AAV9-CAGGS-*GALC* to achieve extension of lifespan to 9 months with long-term improvement of neurological and physiological signs (Marshall et al., 2018). Considering these data, recent findings that ICM administration of high dose AAV9-CAGGS-GALC (1x10^14^ vg) prior to disease onset in a naturally occurring canine model for Krabbe disease decreased psychosine concentration and inflammation, improved myelination, nerve conduction and extended lifespan from 16 weeks to at least 2.5 years are very promising (Bradbury et al., 2020). In contrast, combined IV and ICV AAV.rh10-CAG-*GALC* at a lower dose (3.8x10^13^ vg) while ameliorating CNS and PNS disease only doubled the lifespan (Bradbury et al., 2018). Recent promising results were observed upon combination of high dose IV of AAVrh10 – mGALC (1.6 x 10^14^ vg/kg) with bone marrow transplant between P10 – P11 in twitcher mice extended median survival over 500 days (Karumuthil-Melethil et al., 2016; Ricca and Gritti, 2016; Pan et al., 2019; Rafi et al., 2020). Although the rapid disease progression and limited peripheral involvement warrant an AAV mediated approach, therapeutic outcome is likely to be further improved by combination with next generation HSC-GTs that have addressed the pitfall of selective GALC toxicity in the hematopoietic compartment by introducing a mir126 target sequence in the LV GALC expression vector, without affecting HSC engraftment and monocyte derived macrophage mediated GALC expression in the CNS (Ungari et al., 2015). In a separate murine study, better GALC secretion and BBB transfer was achieved by attaching the iduronate-2-sulfatase signaling peptide and the apolipoprotein B low-density lipoprotein receptor to the GALC coding sequence improving myelination, motor function and life span compared to previous studies (Pan et al., 2019).

Based on studies in mice the vulnerability to *GalC* ablation appears to be highest between P4 and P6 before onset of myelination, and *GalC* expression is highest in OPCs, newly formed oligodendrocytes and astrocytes, but drops in mature oligodendrocytes (Weinstock et al., 2020). This indicates that ensuring widespread transduction and gene expression in OPCs, neural crest cells as well as early myelinating oligodendrocytes and Schwann cells which are most in need of GALC may be important and should be addressed during design of the next generation of Krabbe disease targeting gene therapies. Notably, aside from the *MAG*, the *CNP* and the *Mbp* promoter, in context of AAV9 the CBh promoter showed strong functional transduction in oligodendrocytes but it remains to be determined if this includes OPCs and early myelinating oligodendrocytes (Powell et al., 2020). The same holds true for the oligodendrocyte targeting capsid variant Olig001 and the neural stem cell targeting AAV.SCH9.

Krabbe disease pathology demands rapid onset and strong GALC expression and thus scAAV genomes may be best suited and the synthetic Olig2 derived promoters Ple266 and Ple267, or the 0.3 kb *MAG* promoter may be candidates; bearing in mind that some astroglial expression could prove beneficial. Lastly, even when combined with HSC-GT, an AAV vector capable of highly efficient transcytosis through the CBB or BBB with reduced off target transduction would be pivotal.

## Leukodystrophies That Require Astrocyte Targeting

Astrocytes are the most abundant cell type in the human brain where they control perivascular homeostasis, integrity of the blood brain barrier and provide trophic support of neurons and oligodendrocytes. In addition, astrocytes are key to neurotransmitter recycling and directly modulate synaptic activity (Santello et al., 2019; Sweeney et al., 2019; Cohen-Salmon et al., 2020; Nutma et al., 2020). In white matter tracts, astrocytes and oligodendrocytes are directly connected through gap junctions formed by connexin hemichannels, generating a panglial syncytium with ependymal cell that enables free flow of water and small molecules, including lactate, amino acids, gliotransmitters and ions. Astrocytes are thought to prevent intramyelinic oedema by diverting excessive osmotic water arising in paranodal myelin from action potential associated ion fluxes. Astrogliopathies caused by mutations in genes coding for astrocytic connexin hemichannels including Cx43 and Cx30 required for gap junction formation with oligodendrocytes and in genes encoding proteins associated with ion-water homeostasis, frequently present as vacuolating leukodystrophies (Lutz et al., 2009; Tress et al., 2012; van der Knaap and Bugiani, 2017). In addition, iron supply provided by astrocytes is essential for oligodendrocyte metabolism and myelination (Nutma et al., 2020). Reactive astrogliosis in the brain induced by transient stressors or progressive neurodegeneration generally contributes to restoration of metabolic homeostasis and overall neuroprotection and repair, but may be detrimental if the driver persits (Garcia et al., 2020). In some leukodystrophies the underlying genetic cause is originates from mutations of astrocyte specific genes and their monogenic cause, disease severity and absence of alternate treatments puts these leukodystrophies in scope for gene therapy development.

### Alexander Disease

Alexander disease (AxD) is caused by autosomal dominant *gain of function* mutations in the gene encoding the intermediate filament glial acidic fibrillary protein (*GFAP*). In its severe infantile presentation AxD is characterized by megalocephaly, hypomyelination, developmental delay, psychomotor decline and seizures culminating in premature demise (Prust et al., 2011; Köhler et al., 2018). The brain pathophysiology involves a toxic accumulation of the mutated GFAP protein in fibrous multiprotein complexes with ubiquitin, vimentin, heat shock protein HSP27, plectin and αβ-crystallin in eosinophile complexes termed Rosenthal fibers. AxD astrocytes are characterized by a bushy, activated morphology, with decreased glutamate buffering activity and an inhibitory effect on oligodendrogenesis and myelination (Brenner et al., 2001; Lanciotti et al., 2013; Brignone et al., 2015; Olabarria and Goldman, 2017; Li et al., 2018).

Since the disease is caused by toxic accumulation of a mutant misfolded protein and has predominantly neurological manifestation, gene therapy should be CNS targeted and aim to reduce the expression of mutant GFAP in astrocytes. This may be achieved through AAV – mediated expression of a GFAP targeting synthetic miRNA, preferentially incorporated into an astrocyte enriched primary miRNA backbone (Jovièiæ and Gitler, 2017) and driven by an astrocyte specific promoter. If the miRNAs’ complementary target sequence is located in the 5’-UTR or 3’-UTR of the *GFAP* mRNA, simultaneous expression of transgenic wt *GFAP* is feasible. In a different approach, a single intrathecal injection of a GFAP mRNA targeted ASO with enhanced nuclease resistance, binding affinity and lower toxicity has achieved remarkable efficacy providing elimination of GFAP throughout the CNS, reversal of Rosenthal fibers and rescue of hippocampal neurogenesis in an AxD mouse model (Hagemann et al., 2018).

### Van der Knaap Disease

Autosomal recessive mutations in Megalencephalic Leukoencephalopathy with subcortical cysts (*MLC)1*, or dominant heterozygous mutations in *GLIALCAM* disrupt membrane localization of MLC1 to astrocyte endfeet and astrocyte junctions, where it stabilizes water channels including aquaporin-4 and the inwardly rectifying potassium channel Kir4.1 (Noell et al., 2011; Brignone et al., 2015). Disruption of the ion-water homeostasis and cell volume regulation in van der Knaap disease is thought to be the key pathophysiological hallmark leading to this spongiform leukodystrophy that morphologically manifests with megalocephaly and intramyelinic vacuolization. The more common infantile form is characterized by megalencephaly caused by chronic white matter oedema. In the classical progressive form of the disease, loss of motor functions, epilepsy, and mild mental decline follow, and patients are often wheelchair – bound by their teenage years. Interestingly, dominant mutations in *GLIALCAM* have been shown to cause a remitting form of Van der Knaap disease.

MLC1 is exclusively expressed in gray and white matter astrocytes including cerebellar Bergmann glia in the CNS. In a recently published murine MLC1 knockout model that mirrors the spongiform phenotype of the human disease, a single astrocyte targeted AAV.rh10-*GFAP-MLC1* gene replacement prevented vacuolization when administered before disease onset at 5 months, and even at 15 months provided near complete remission of vacuolization (Sánchez et al., 2020). While likely due to the advanced age at infusion, the authors observed relatively poor transduction in the CNS following IT or ICV delivery. Interestingly, AAV delivery into the intracerebellar subarachnoid space provided more efficient and widespread transduction throughout the cerebellum compared with deeper injections into the white matter or molecular layer. Because this delivery route does not require physical penetration deep into the CNS it is less invasive and appears worth further investigation as an alternative or additional AAV delivery route for many leukodystrophies and leukoencephalopathies.

Two further albeit later onset and usually milder leukodystrophies may benefit from astroglial gene transfer include chloride voltage gated channel 2 (*CLCN2*) related vacuolating leukoencephalopathy and the Oculodentodigital dysplasia with cerebral white matter abnormalities (ODDD), a hypomyelinating leukodystrophy caused by an autosomal dominant mutation in the Gap junction α 1 (Connexin-43) encoding *GJA1* gene (Abrams and Scherer, 2012). Both genes are predominantly expressed in astrocytes in mice and humans. Given that using current vectors the efficiency of ICV, ICM and IV gene delivery rapidly decreases with age, development of better BBB and CBB penetrating capsids is required.

## Microglia as a Target for Leukodystrophy Treatments

Microglia are the only hematopoietic stem cell derived cell type in the healthy CNS, where they are considered to be the resident macrophages. They are ontogenically distinct from other mononuclear phagocytes, including dendritic cells, monocytes and macrophages. They originate from the yolk sac and infiltrate the developing CNS where they compose ∼10% of all glial cells and self – maintain. Microglia are highly dynamic and constantly survey their microenvironment by active projection and retraction and are now known to perform much more than immune – related functions in the CNS (Wu et al., 2015). Over the past decade microglia have been shown to actively participate in synapse remodeling and stripping, as well as to actively maintain or compromise the integrity of the BBB (Wake et al., 2009; Akiyoshi et al., 2018; Haruwaka et al., 2019). Nevertheless, in their crucial role as immune cells, upon insult, infection or neural cell degeneration microglial rapidly change from a highly ramified to a more amoeba-like shape, proliferate and express a range of pro-inflammatory cytokines. With regards to leukodystrophies, microglia have become both a target in case of certain microgliopathies and a therapeutic tool as they are critical to clearance of myelin debris, a prerequisite for remyelination.

In a few leukodystrophies classified as microgliopathies, mutations in microglia specific genes are the root cause of the disease. These include colony stimulating factor 1 receptor (CSF1R) associated ‘’hereditary diffuse neuroaxonal leukoencephalopathy with axonal spheroids’ and the ‘pigmentary orthochromatic leukodystrophy’ (Rademakers et al., 2011; Nicholson et al., 2013) as well as Nasu – Hakola disease caused by *DAP12* gene mutations encoding the TYRO protein tyrosine kinase binding protein (Sasaki, 2017). These microgliopathies are usually adult onset leukodystrophies exhibiting neurological phenotypes as well as peripheral manifestations. Although microglia self-renew locally, under certain disease conditions or following myeloablation, circulating monocytic precursor cells can infiltrate the CNS and differentiate along the macrophage pathway into microglia-like cells and perform microglial functions (Greter et al., 2015; Beins et al., 2016). This mechanism in combination with the close ontogenic relationship indicates that some microgliopathies may be at least partially amendable by *ex vivo* HSC-GT.

Interestingly, RNAseq data assessing cell type – specific gene expression profiles in mice (Table 2) demonstrates that many LSDs associated genes are expressed at comparably high levels in microglia and microglial damage has been shown to precede myelin damage in MLD and X-ALD (Bergner et al., 2019). Engraftment of autologous bone marrow or cord blood derived genetically ‘corrected’ HSCs expressing a functioning copy of the mutated gene are becoming a viable treatment option for some leukodystrophies. Following myeloablation between 5 and 20% of microglia are physically and functionally replaced with transgenic, HSC – derived microglia-like macrophages capable of cross-correction through lysosomal enzyme secretion or intracellular processing of substrate that would otherwise accumulate to toxic levels.

*Ex vivo* HSC-GT has shown immense promise halting disease progression when performed in pre-symptomatic or early symptomatic patients and is currently in various stages of clinical trial for lysosomal and peroxisomal storage disorders including MLD and X-ALD disease, (Dahl et al., 2015; Nagree et al., 2019). The first HSC-GT, for a CNS disease ‘Libmeldy’, has just been approved by the European Commission for use in children with late-infantile onset MLD. MLD is an autosomal recessive demyelinating leukodystrophy caused by of arylsulfatase A (*ARSA*) deficiencies triggering an accumulation of the myelin lipid sulfatide in oligodendrocytes and Schwann cells, as well as several peripheral organs, because the enzyme can no longer support its degradation and recycling. In this most common and severe late-infantile form of the disease, children lose the ability to walk and talk within months, and following rapid progression of motor symptoms, commonly die within seven years of diagnosis making the success of ‘Libmeldy’ a truly remarkable testament to the field. Pioneering the technology, HSC-GT successfully halts disease progression in X-ALD, even though in this case the peroxisomal very long chain fatty acid transporter ABCD1 is not secreted. In the STARBEAM study, HSC-GT for X-ALD is now also in late-stage Phase II/III clinical trial (NCT01896102, NCT02698579, NCT02559830). Recent success in early symptomatic treatment of adult-onset X-ALD using HSCT underscores the potential feasibility of this approach for adult-onset LSDs and the above mentioned microgliopathies (Matsukawa et al., 2020).

Other notable LSDs that manifest with neurological symptoms, frequently present with white matter abnormalities, and may benefit from HSC-GT include Tay-Sachs disease from monosialic ganglioside accumulation following mutation in the hexosaminidase A (*HEXA*) gene, Sandhoff disease associated with HEXB mutations and Sanfilippo syndrome, a mucopolysaccharidosis (MPSIII) stemming from deficiencies in α-N-acetylglucosaminidase (*NAGLU*) or N-sulfoglucosamine sulfohydrolase (*SGSH*) gene. Recent development of a bicistronic lentivirus *HEXA* and *HEXB* encoding vector promoting stoichiometric synthesis of both subunits of the enzyme is capable of targeting both Tay-Sachs and Sandhoff disease in HSC-GT (Ornaghi et al., 2020). Interestingly, α – mannosidase encoding MAN2B, mutations in which cause α – mannosidosis, is also predominantly expressed in microglia, indicating that this lysosomal storage disease may also benefit from HSC-GT, and potentially add to recent achievement in AAV mediated preclinical success in a feline model (Zhang et al., 2014; Yoon et al., 2020).

A pitfall of *ex vivo* gene therapy for leukodystrophies and leukoencephalopathies is the crucial importance for early treatment; arrest of disease progression in X-ALD took 12-18 months in which demyelinating lesions continued to expand (Cartier et al., 2014). Although myeloablation efficiently creates a niche and HSC engraftment occurs within hours to days, functional microglia – like differentiation increases over months after treatment with superior efficacy of ICV over IV HSC administration recently reported (Capotondo et al., 2017). Aside from engraftment and differentiation additional factors including transduction efficiency and transgene expression may contribute to the observed delay in halting often irreversible disease progression. A recent notable advance that may drastically improve the outcome of HSC-GT demonstrated that targeted inhibition of the endogenous microglial proliferation through inhibition of CSF1R effectively before myeloablation enabled near complete engraftment of (>92%) transgenic microglial like cells (Xu et al., 2020).

Separately the pitfall of a delayed effectiveness of HSC-GT for treatment of some neurometabolic disorders may be overcome by gene replacement through rAAV because it yields expression of a missing gene within days in case of scAAV, or weeks for ssAAV counterparts. Indeed, clinical trials employing broad spectrum AAV vectors with transgene expression driven by ubiquitous promoters are currently underway for MLD, Tay – Sachs disease and Sandhoff disease, as well as Sanfilippo syndrome in which both direct intraparenchymal delivery of ssAAVs (NCT03612869) and systemic delivery of scAAV9 (NCT02716246) are investigated. A recently initiated phase I dose escalation study for Tay – Sachs and Sandhoff disease (NCT04669535) combines dual ICM and IT administration of AAV.rh8-HexA and AAV.rh8-HexB at a 1:1 with bilateral intrathalamic delivery.

Looking forward, bicistronic HexA and HexB which can circumvent dual AAV delivery have shown promise in murine models of Sandhoff disease (Lahey et al., 2020). To date, the AAV – mediated gene therapies selectively targeting microglia have been hampered by poor transduction efficiencies (<20 %) *in vivo*. However, the success of HSC-GT in combination with the observation that microglial damage precedes myelin damage indicate that development of therapeutic AAV vectors with better microglial tropism may promote therapeutic outcomes even for leukodystrophies like MLD that may require a broader cell type overlapping approach.

## Leukodystrophies Requiring Targeting of Multiple Cns Cell Types

Based on the relatively broad expression and the ubiquitous requirement of the gene product, a number of leukodystrophies caused by ‘loss of function’ that do not result in the accumulation of a toxic metabolite are likely to require broad cell type overlapping gene replacement. Their gene products are frequently part of multiprotein complexes, disease severity and onset are variable and additional pathologies in the PNS and peripheral organs are often involved. Many of these enzymes are ubiquitously expressed, and absence or complete loss of function are usually embryonically lethal. Examples include autosomal recessive aminoacyl transfer RNA (tRNA) related leukodystrophies, ‘hypomyelination with brainstem and spinal cord involvement and leg spasticity’ (HBSL) caused by mutations in the cytosolic aspartyl-tRNA synthetase 1 (*DARS1*) (Taft et al., 2013; Fröhlich et al., 2017, 2018), ‘leukoencephalopathy with brain stem spinal cord involvement ad lactate elevation’ (LBSL) associated with mitochondrial *DARS2* mutations (van der Knaap et al., 1995; Scheper et al., 2007), the cytosolic and mitochondrial alanyl-tRNA synthetase (*AARS1, AARS2*) underlie progressive ‘leukoencephalopathy with ovarian failure and epileptic encephalopathy 29’ (Dallabona et al., 2014; Simons et al., 2015), mutations in’ glutamyl-tRNA synthetase (*EARS1*) cause ‘leukoencephalopathy with thalamus and brainstem involvement and high lactate’ (LTBL) (Steenweg et al., 2012), arginyl-tRNA synthetase (*RARS1*) cause ‘hypomyelinating Leukodystrophy 9’ (Mendes et al., 2020) and the aminoacyl-tRNA synthase complex-interacting multifunctional protein 1 (AIMP1) (Feinstein et al., 2010). Similarly, ‘4H-leukodystrophy’ originates from mutations in RNA polymerase 3 (*POLR3A* and *POLR3B*) encoding genes and is typically characterized by hypomyelination, hypodontia, and hypogonadotropic hypogonadism. Age of onset varies but averages four years of age and *POL3A* mutations are associated with more severe disease. POLR3A and POLR3B are the main building blocks of this RNA polymerase that transcribes tRNAs and ribosomal RNA (rRNA) (Wolf et al., 2014; Thiffault et al., 2015; van der Knaap and Bugiani, 2017). To date discovery of adequate animal models that enable preclinical testing of therapeutic strategies is ongoing, however gene therapies will likely need to reflect the broad expression profile of these genes.

Aicardi-Goutieres Syndrome (AGS) is caused by mutations in a number of genes involved in nucleotide metabolism. These include *RNASEH2A, RNASEH2B RNASEH2C.* AGS manifests as microcephaly with progressive spasticity, psychomotor retardation. Neonatal onset has been associated with an approximately 35% fatality in early childhood. RNASEH2 is a DNA repair enzyme and its absence leads to DNA damage response and accumulation of cytosolic DNA aggregates triggering inflammation. While expressed across neural cell types, astrocytes rather than infiltrating leukocytes were found responsible for high cytokine and particularly interferon-α production in post-mortem brains indicating that gene replacement therapies, while targeting all cell types, should first and foremost ensure good astroglial coverage, but not prevent expression in other cells. Either way, a gene therapy for RNASEH2 related leukodystrophies must be carefully designed and tested because the enzyme consists of three albeit small subunits and to provide constraints around stoichiometry will be challenging. In addition, it should be noted that *RNASEH2C* is considered a metastasis susceptibility gene and interfering with the expression of cell cycle regulated genes must be very carefully considered as it may increase downstream risks of malignancies (Crow et al., 2006; Orcesi et al., 2009; Crow and Manel, 2015; Deasy et al., 2019).

Lysosomes play essential roles in the turnover of structurally diverse compounds including lipids, proteins, nucleic acids and oligosaccharides and thus control homeostasis through the autophagy – lysosomal pathway. Mutations in many genes associated with these pathways cause lysosomal storage diseases (LSD) characterized by the accumulation of intermediate metabolites that are frequently toxic at higher concentrations and prevent homeostasis, eventually triggering cell death. Many LSDs have significant neurological and CNS involvement. While cross-correction by externally delivered lysosomal enzymes can mitigate peripheral aspects of the pathology, in the CNS uptake is largely prevented by the BBB, promoting the development of gene therapies that have the potential to overcome limitations of traditional enzyme replacement therapies. We apologize to our colleagues in this field whose valued contributions could not be included in this manuscript due to space constraints. Recent reviews have provided comprehensive overviews on clinical progress of gene therapies (Hocquemiller et al., 2016; Nagree et al., 2019; Parenti et al., 2021). Aside from the above mentioned MLD, Krabbe disease, Tay-Sachs and Sandhoff disease, GM1 gangliosidosis is an autosomal recessive LSD associated with progressive neuronal cell death in the brain and spinal cord caused by mutations in the β-galactosidase encoding gene *GLB1* that is expressed by all cell types in the CNS (Brunetti-Pierri and Scaglia, 2008). Reflecting the rapid neurological decline associated with neuron loss in GM1 gangliosidosis, early, ideally presymptomatic treatment is essential. Likely due to insufficient enzyme expression in the CNS a trial of HSCT could not prevent neurological disease progression (Shield et al., 2005). In contrast, following promising results in preclinical research in murine and feline animal models employing AAV in which GLB1 expression was driven by strong constitutive promoters (Broekman et al., 2007; Weismann et al., 2015; Gray-Edwards et al., 2017; Hinderer et al., 2020), intravenous AAV9 (NCT03952637), ICM administration of AAVhu.68 (NCT04713475) and AAVrh.10 (NCT04273269) have advanced into clinical trial. Further, multiple sulfatase deficiency (MSD), a similar lysosomal storage – related leukodystrophy with significant CNS involvement arising from autosomal recessive mutation in the sulfatase-modifying factor 1 (SUMF1) encoding gene, causes profound reduction in downstream sulfatase activities, including the sulfatases associated with other LSDs like MLD and various mucopolysaccharidosis (MPS) (Fraldi et al., 2007). MSD patients display combined clinical symptoms of these sulfatase deficiencies, with severity depending on the specific mutation (Schlotawa et al., 2011). In LSDs disease onset is inversely with correlated neurological decline and severity. To prevent neurological deterioration rapid and widespread *SUMF1* expression and activity is paramount. Combined ICV and IV infusion of AAV9 -CMV-SUMF1 was able to activate sulfatases, clear accumulated glycosaminoglycans, decrease inflammation and improve motor and memory performance (Spampanato et al., 2011). In future, scAAV vectors promoting quicker onset of lysosomal enzyme expression following IP, ICM or systemic IV delivery in LSDs are likely to emerge.

### Vanishing White Matter Disease

VWM disease, also known as ‘childhood ataxia with central nervous system hypomyelination’ is a prominent leukodystrophy that is caused by loss of function in eukaryotic translation initiation factor 2B subunit 1 to 5 (*EIF2B1, EIF2B2, EIF2B3, EIF2B4, EIF2B5*) encoding genes, that show a clear genotype – phenotype correlation. The nucleotide exchange factor eIF2B is required for eIF2 delivery of initiator Met-tRNA to the ribosome and initiation of translation (Leegwater et al., 2001). Classical VWM disease is associated with progressive neurological deterioration, mild spasticity, and cognitive impairment. Stress causes episodes of major deterioration that may be fatal. Indeed, eI2FB is the critical regulator of the integrated stress response during protein translation and is activated by oxidative stress or starvation (Carter, 2007). Based on this crucial role, EIF2B appears fairly uniformly expressed across CNS cell types (Table 2) but the most striking histopathological changes in VWM are observed in astrocytes and oligodendrocytes.

Recent evidence from a glial progenitor cell therapy study suggested that phenotypic improvement is correlated with increased astroglial differentiation underscoring the hypothesis that astrocytes are central to the VWM pathology (Dooves et al., 2019). In addition, co – cultures of mutated astrocytes with wildtype oligodendrocytes impaired maturation of the oligodendrocytes, while mutated oligodendrocytes mature normally in the presence of wildtype astrocytes (Dooves et al., 2016). These findings indicate that primary gene replacement must also first and foremost target astrocytes. However, the broad expression of EIF2B1 – 5 across different cell types in the CNS indicates, broad cell type targeting within the CNS will be required. All *EIF2B* genes are expressed at high levels throughout peripheral organs and peripheral phenotypes such as endocrine effects have been observed and may also need to be addressed. Nevertheless, the pre-clinical benefits achieved through glial progenitor cell therapy are encouraging. Since most AAV vectors that penetrate the BBB or CBB transduce astrocytes with high efficacy following IV or ICM administration, AAV gene replacement with constitutively active promoters appears to be a viable treatment option for severe infantile VWM disease. For treatment development, capsid and promoter selection would certainly benefit from a screening for ‘functional transduction’ in a humanized animal model.

## Conclusion and Future Directions

Nucleic acid-based treatments of neurological disorders have rapidly expanded in the last decade and the long – recognized potential for gene therapy is now becoming a reality for patients and families suffering from a multitude of devastating genetic diseases. The first hematopoietic stem cell-based *ex vivo* gene therapies as well as AAV – mediated *in vivo* gene therapies have been approved by the FDA. In addition, the first ASOs have also successfully advanced to this stage. In addition, most big pharma companies are supporting development and increasingly managing to address issues associated with large scale production. Nevertheless, despite the immense potential and undoubtedly growing importance of these evolving genetic drugs in modern medicine, recent clinical and pre-clinical studies also expose risks and pitfalls underscoring the importance of a cautious and considered advance. In the case of *ex vivo* HSC-GT, this has ensured development of self-inactivating lentiviral vectors and rigorous mapping of integration sites. With regards to AAV – mediated *in vivo* gene therapy programs, which are establishing excellent safety and efficiency profiles, the recent tragic deaths of three patients in the high dose cohort of the ASPIRO trial targeting myotubular myopathy as well as preclinical evidence of toxicity and severe immune response associated with high vector doses following systemic AAV delivery are a reminder of the risks and imperative for refinement, particularly around vector dose. This is of particularly relevant to treatments for leukodystrophies and leukoencephalopathies that rely on relatively high vector doses associated with targeting the entire CNS. Since direct intraparenchymal injections are associated with risk of hemorrhage and infection and it has been estimated that about 100 injection sites may be required to achieve full CNS coverage, the discovery of BBB and CBB traversing AAV serotypes has moved systemic IV and CSF vector administration into focus. Importantly, comparison of delivery routes and modes of rAAV vector administration is ongoing, but recent studies indicate when administered into the CSF a substantially better vector spread throughout the CNS and spinal cord is achieved by injection into the cisterna magna over other cerebroventricular and intrathecal approaches. A key to dose reduction is to increase this ability in the next generation of AAV vectors while ensuring immune – evasion and substantial reduction in off-target tissue transduction. Among others, notable preclinical progress has been made in this field through discovery of novel naturally – occurring AAV variants, alongside rational capsid design, capsid shuffling peptide insertion combined with high throughput screening and targeted selection to extend the repertoire of vectors and associated cell and tissue targeting profiles.

By reviewing the natural expression profile of genes associated with leukodystrophies across neural cell types, we hope to provide a better understanding of which cells may naturally be important targets. Highlighting the importance of the choice of promoters and *cis*-regulatory elements to achieve gene expression in the target cell we compiled current capsid and promoters that may be considered as a starting point with regards to development of improved targeting strategies and potentially toggleable expression control. Notably, recent findings that direct capsid – promoter interactions determine the cell type – selective gene expression in the CNS highlight the importance of screening these aspects in conjunction. Because beyond uptake, capsid components influence intracellular transport, stability and gene expression, and cross species differences are evident even when targeting the same cell type, it will be increasingly important to confirm functional transduction in humanized CNS models. AAV capsid / promoter combinations identified via screens resulting from selective pressure for functional transduction of the target cell in humanized models may be a promising strategy to achieve the needed vector dose reduction required for treatment of CNS disorders. Beyond vector engineering and choice, other patient specific, parameters in addition to body weight may need to be considered when choosing vector dose. These include developmental stage, disease progression, immunity, and metabolic profile. As gene therapies for leukodystrophies and leukoencephalopathies advance, the programs will need to broaden to assess and mitigate risk. In many, if not most cases, a single shot ‘Magic Bullet’ curative treatment may be elusive, but such challenges are driving the rapid advance in sophistication of the vectors and therapeutic payloads to yield genetic medicines to treat these devastating neurological diseases.

## Author Contributions

GJ led the project and wrote the draft manuscript. GJ, CR, and GH contributed to the manuscript preparation. All authors read and approved the final manuscript.

## Conflict of Interest

The authors declare that the research was conducted in the absence of any commercial or financial relationships that could be construed as a potential conflict of interest.
